# Barriers and Enablers to Early Identification, Referral and Access to Geriatric Rehabilitation Post-Hip Fracture: A Theory-Based Descriptive Qualitative Study

**DOI:** 10.1177/21514593211047666

**Published:** 2022-03-04

**Authors:** Chantal Backman, Anne Harley, Steve Papp, Veronique French-Merkley, Paul E Beaulé, Stéphane Poitras, Johanna Dobransky, Janet E Squires

**Affiliations:** 1 School of Nursing, Faculty of Health Sciences, 6363 University of Ottawa, Ottawa, Canada; 2 Ottawa Hospital Research Institute; 3 Bruyère Research Institute; 4 Attending Physician in Geriatric Rehabilitation at Bruyere Continuing Care, Faculty of Medicine, 6363 University of Ottawa, Ottawa, Canada; 5 Clinical Director and Trauma Surgeon at The Ottawa Hospital, Faculty of Medicine, 6363 University of Ottawa, Ottawa, Canada; 6 Department Chief in Care of the Elderly at Bruyere Continuing Care, Faculty of Medicine, 6363 University of Ottawa, Ottawa, Canada; 7 Head of the Division of Orthopaedic Surgery at The Ottawa Hospital; Professor Faculty of Medicine, 6363 University of Ottawa, Ottawa, Canada; 8 School of Rehabilitation Sciences, Faculty of Health Sciences, University of Ottawa; 9 Clinical Research Program Manager, Division of Orthopaedic Surgery, 6363 The Ottawa Hospital, Ottawa, Canada

**Keywords:** transitions, hip fracture, geriatric rehabilitation

## Abstract

**Background:**

Geriatric hip fracture patients often experience gaps in care including variability in the timing and the choice of an appropriate setting for rehabilitation following hip fracture surgery. Many guidelines recommend standardized processes, including timely access of no later than day 6 to rehabilitation services. A pathway for early identification, referral and access to geriatric rehabilitation post-hip fracture was created to facilitate the implementation. The study aimed to describe the barriers and enablers prior to the implementation of this pathway.

**Methods:**

We conducted a qualitative descriptive study consisting of semi-structured interviews with geriatric hip fracture patients (n = 8), caregivers (n = 1), administrators (n = 12) and clinicians (n = 17) in 2 orthopaedics units and a geriatric rehabilitation service. Responses were analysed using a systematic approach, and overarching themes describing the barriers and enablers were identified.

**Results:**

The clinicians’ and administrators’ top barriers to implementation of the pathway were competing demands (n = 24); lack of bed availability, community resources and funding (n = 19); and the need for extended hours and increased staff (n = 16). The top 3 enablers were clear communication with patients (n = 27), awareness of the benefits of geriatric rehabilitation (n = 24) and the need for education and resources to properly use the pathway (n = 15). Common barriers among patients and caregivers included lack of care coordination, overcoming some of their own specific challenges during their transition, gaps in the information they received before discharge, not knowing what questions to ask and lack of resources. Despite these barriers, patients were generally pleased with their transition from the hospital to geriatric rehabilitation.

**Conclusion:**

We identified and described key barriers and enablers to early identification, referral and access to geriatric rehabilitation post-hip fracture. These influencing factors provide a basis for the development of a standardized pathway aimed at improving access to rehabilitative care for geriatric hip fracture patients.

## Contributions to the Literature

- This research identified and described implementation barriers and enablers specific to the referral process of post-operative hip fracture patients from acute care to subacute care.
- The barriers and enablers identified will help to improve referral rates to rehabilitation and help to reduce the acute care length of stay with the goal of helping hospitals meet best practice targets.
- A better understanding of these barriers and enablers can inform and optimize future implementation strategies so that they are better tailored to the local context and can also inform wider implementation.

## Background

Hip fractures often represent sentinel events in the health trajectories of frail and chronically ill individuals, precipitating a steep functional decline and permanent disability.^1^ In the United States, the cost of hip fracture care is estimated at more than US$5 billion annually across multiple sectors with most of the cost incurred in the post-acute care setting.^2–5^ As a result, the economic burden of geriatric patients with hip fractures causes significant and unnecessary strain on the health care system.

Many initiatives have been developed worldwide to improve patient outcomes and reduce cost.^6–8^ The American Geriatrics Society (AGS) and the International Geriatric Fracture Society (IGFS) have identified the best available evidence for post-acute care settings to improve the outcomes of geriatric hip fracture patients, as well as minimize complications.^9^ In addition, many guidelines recommend all patients with hip fracture receive active rehabilitation following their acute care stay with rehabilitation beginning no later than 6 days following surgery.^1,10–12^

Nonetheless, geriatric hip fracture patients often experience gaps in care including variability in the timing and the choice of the appropriate setting for rehabilitation following hip fracture surgery. Inpatient geriatric rehabilitation is recommended as the gold standard for post-hip fracture care when striving to maximize functional recovery.^13^ Geriatric rehabilitation involves a set of multidisciplinary interventions with the aim of restoring functional ability and enhancing residual functional capability in older adults with disabling impairments.^14,15^ Several studies have highlighted the diversity in post-acute care pathways that exist.^16–18^ According to Pitzul and colleagues,^16^ there are pervasive variations in post-acute care delivery for geriatric hip fracture patients, especially with respect to access to rehabilitation.^16^ In their study, the researchers identified over 49 unique post-acute care pathways taken by hip fracture patients to access rehabilitation. Results showed that the flow of hip fracture patients into geriatric rehabilitation is generally inconsistent. Furthermore, they concluded that a treatment model for geriatric hip fractures should emphasize the need to implement standardized plans of care, should work with all members of the health care team and should provide for continual quality improvement.^16^

Research shows that timely access to rehabilitation services following hip fracture surgery results in better patient outcomes.^19,20^ Our institution currently faces substantial variations in referral rates to rehabilitation and also in acute care length of stay. Our current transition from acute to subacute care occurs at an average of 12.7 days which is above the recommendation of no later than 6 days post-surgery.

To address the complexities of the timely referral of hip fracture patients to geriatric rehabilitation, our research’s overall purpose was to develop and implement a theory-based intervention for an earlier discharge of the geriatric hip fracture population transitioning from acute care to subacute care. As such, the purpose of our study was to describe the barriers and enablers to early identification, referral and access to geriatric rehabilitation post-hip fracture.

## Methods

### Study Design and Setting

In this qualitative descriptive study, we conducted semi-structured interviews between 2018 and 2019, with geriatric hip fracture patients, informal caregivers, administrators and clinicians on 2 orthopaedics units in a large academic health sciences centre and on a geriatric rehabilitation service in an academic health care organization. The research ethics board’s approval was obtained.

### Participant Eligibility

We used a purposive sampling technique to recruit clinicians involved at different stages of the referral pathway. This included any physicians (surgeons, geriatricians and internal medicine), nurses, physiotherapists, social workers, transition care coordinators and occupational therapists involved in the management of hip fracture patients either on the orthopaedics units or on the geriatric rehabilitation service. For the patients and informal caregivers, we used a convenience sampling method to recruit hip fracture patients (> 65 or older) and their informal caregivers (> 18 or older). Participants were recruited until saturation was obtained.

### Data Collection

For the clinicians’ interviews, we designed an interview guide informed by the Theoretical Domains Framework.^21–23^ The Theoretical Domains Framework (TDF) is a robust and integrative theoretical framework, developed by health psychologists and health services researchers, based on a synthesis of 33 behaviour change theories, clustered into 14 theoretical domains.^21,22^ The TDF was developed for implementation research across multiple disciplines and is often used in behaviour change research.^21–23^ The framework was validated with behaviour change experts and has been used to investigate barriers and enablers to intervention implementation in a variety of clinical situations. A list of sample interview questions for each of the 14 TDF domains is provided in Table 1.

**Table 1.** Clinicians’ Sample Interview Questions for Each of the 14 TDF Domains.

Domain	Definition [12]	Sample Interview Question
Knowledge	An awareness of the existence of something	Are you aware of any guidelines or practices regarding the management of geriatric (> 65) hip fracture patients transitioning from acute care to rehabilitation?
Skills	An ability or proficiency acquired through practice	What skills, experience or specific training are needed for the management of geriatric hip fracture patients transitioning from acute care to rehabilitation?
Social/professional role and identity	A coherent set of behaviours and displayed personal qualities of an individual in a social or work setting	Is there anything about your professional role (as a nurse/physician/other) that influences your approach to managing geriatric hip fracture patients transitioning from acute care to rehabilitation (prompt: Special training, a protocol, other technologies)?
Beliefs about capabilities	Acceptance of the truth, reality or validity about an ability, talent or facility that a person can put to constructive use	As a physician/nurse/other, how confident are you in your ability to properly decide if a patient is appropriate for the pathway?
Optimism	The confidence that things will happen for the best or that desired goals will be attained	Do you think a streamlined pathway including earlier identification, referral and access to geri-rehab post-hip fracture would help support geriatric hip fracture patients to transition from acute care to rehabilitation? If yes, how? If no, why not?
Beliefs about consequences	Acceptance of the truth, reality or validity about outcomes of a behaviour in a given situation	How easy or difficult is it to begin or follow the process for managing geriatric hip fracture patients transitioning from acute care to rehabilitation?
Reinforcements	Increasing the probability of a response by arranging a dependent relationship or contingency between the response and a given stimulus	What would make it easier or would be an incentive in your practice or for you personally to use a streamlined pathway to support geriatric hip fracture patients transitioning from acute care to rehabilitation?
Intentions	A conscious decision to perform a behaviour or a resolve to act in a certain way	Do you intend to use a streamlined pathway to support geriatric hip fracture patients transitioning from acute care to rehabilitation? If no, why? If yes, do you anticipate any problems?
Goals	Mental representations of outcomes or end states that an individual wants to achieve	How important is it for you to manage geriatric hip fracture patients transitioning from acute care to rehabilitation?
Memory, attention and decision processes	The ability to retain information, focus selectively on aspects of the environment and choose between 2 or more alternatives	Is this process an automatic part of your practice, or do you need to be reminded of it?
Environmental context and resources	Any circumstance of a person’s situation or environment that discourages or encourages the development of skills and abilities, independence, social competence and adaptive behaviour	What aspects of your clinical environment influence whether or not you are able to fully support geriatric hip fracture patients transitioning from acute care to rehabilitation (prompt: Material resources, unit culture, team, events)?
Social influences	Those interpersonal processes that can cause individuals to change their thoughts, feelings or behaviours	Does any other team member influence your approach with the management of geriatric hip fracture patients transitioning from acute care to rehabilitation? If yes, who and how?
Emotion	A complex reaction pattern, involving experiential, behavioural and physiological elements, by which the individual attempts to deal with a personally significant matter or event	What feelings do you experience when you think about the process for managing geriatric hip fracture patients transitioning from acute to subacute care?
Behavioural regulation	Anything aimed at managing or changing objectively observed or measured actions	What do you think is needed to ensure consistent support for the management of geriatric hip fracture patients transitioning from acute care to rehabilitation (individual, team and setting)?

For the patients’ and informal caregivers’ interviews, we developed an interview guide to obtain their views with respect to the management of geriatric patients leaving the hospital for rehabilitation following hip fracture surgery (Table 2).

**Table 2.** Sample Interview Questions for Patients and Informal Caregivers.

Patients in acute and subacute care		1. What is your understanding about rehabilitation after your hip fracture surgery? 2. What are your expectations in regards to leaving the hospital to obtain rehabilitation services after your hip fracture surgery? 3. Were you provided with information you need (or needed) to make a decision about leaving the hospital to go to rehabilitation after your hip fracture surgery? Please explain 4. Was a clear plan explained to you and were all your questions answered? Please explain. (Explore experiences of participants who feel their needs are not being (or were not) met.) 5. How do (or did) you feel about leaving the hospital to go to rehabilitation after your hip fracture surgery? 6. Were you involved as much as you wanted to be in decisions about you leaving the hospital to go to rehabilitation?	
Patients in subacute care only		7. How well coordinated was your transition from the hospital to rehabilitation after your hip fracture surgery? 8. Was there a clear plan? (Probe experiences of fragmentation) Prompts -How well do the people providing your care worked together? -How often did or do you have to repeat information that you had already provided? 9. Are there ways the transition from the hospital to rehabilitation you experienced could be made better? Prompts -What worked? -What did not? 10. If you could change one thing about the transition from the hospital to rehabilitation, what would it be? 11. What did you value most about leaving the hospital to go to rehabilitation? 12. Do have anything else you would like to add or discuss?	

All participants provided signed informed consent prior to being interviewed. After providing consent, the research assistant conducted the digitally recorded 60-minute semi-structured interviews with the patients, the informal caregivers and the clinicians.

### Data Analysis

For the clinicians’ interviews, the transcripts were analysed following a 6-step process as follows^24^: (1) 2 reviewers independently coded the transcripts using the TDF as a coding framework; (2) belief statements were developed for each quote, and then, similar statements were merged; (3) themes were generated from the merged belief statements, allowing similarities and differences to be recognized across the clinician groups; (4) themes were grouped into broader categories; (5) each theme was classified as a barrier or an enabler and (6) themes were examined in relation to whether they were shared (i.e. frequency of specific beliefs across interviews, presence of conflicting beliefs and perceived strength of the belief impacting the behaviour). A third researcher helped to resolve any disagreements. In order for a belief statement to be identified as a barrier or enabler, they had to be shared between at least 2 people or 2 provider groups.

For the patients’ and informal caregivers’ interviews, 2 researchers independently coded the transcribed interviews. The individual analyses were then collectively analysed by the team members for similarities between the transcripts using an iterative process until consensus on the coding and thematic analysis was reached.^25^ NVivo qualitative data analysis software (QSR International Inc,) was used to support the analysis.

### Role of the Funding Source

The funders played no role in the design, conduct or reporting of this study.

## Results

### Participant Characteristics

We interviewed a total of 38 participants, consisting of patients (n = 8), an informal caregiver (n = 1), administrators (n = 12), physicians (n = 7), nurses (n = 2) and other health professionals (n = 8). The other health professionals were physiotherapists (n = 4), occupational therapists (n = 2), social worker (n = 1) and pharmacist (n = 1). The mean age of patient participants was 79.88. Additional characteristics are available in Table 3.

**Table 3.** Participant Characteristics (n = 38).

Characteristic	Patients (n = 8)	Caregiver (n = 1)	Administrators (n = 12)	Physicians^a^ (n = 7)	Nurses (n = 2)	Other Health Professionals (n = 8)
Age						
26–30	n/a	n/a	0	1	0	3
31–40	n/a	n/a	4	2	2	0
41–50	n/a	n/a	4	4	0	4
51–60	n/a	n/a	4	0	0	1
65+	8	1	n/a	n/a	n/a	n/a
Gender						
Male	1	0	0	5	0	1
Female	7	1	12	2	2	7
Location						
Orthopaedics unit #1	2	0	4	3	0	0
Orthopaedics unit #2	2	0	3	2	1	4
Geriatric rehabilitation service	4	1	5	2	1	4
Years of experience on unit						
< 1	n/a	n/a	3	0	0	0
1–5	n/a	n/a	4	2	1	4
6–10	n/a	n/a	1	2	0	1
11–15	n/a	n/a	3	1	1	1
16+	n/a	n/a	1	2	0	2

^a^includes physician assistant.

### Clinicians’ Interviews (n = 29)

The barriers and enablers related to early identification, referral and access to geriatric rehabilitation post-hip fracture were identified. All 14 TDF domains were relevant. A total of 19 belief statements were barriers (across 11 domains), 17 were enablers (across 13 domains) and 4 were conflicting (Tables 4-6).

**Table 4.** Barriers to the Implementation of Best Practices for the Management of Hip Fracture Patients Transitioning from Acute Care to Subacute Care (n = 19).

Category	Overarching barriers across domains	N^a^	%	Domain	Who said it?	Example quote
Workflow	Competing demands (ministry requirements, increased workloads, delays in transitions due to transportation, poor coordination and access to translators)	24	83	Environmental context	Administrators, physicians, nurses and other health professionals	I think there’s different pressures within the system, one pressure is this culture of patient flow and making sure that hospitals are meeting healthy turnaround to be able to have demanded supplies sort of curve met with beds and whatnot. Also, it has a big factor on resource usage and allocation. But I think also the fact... the new culture that comes with the quality- based metrics that are associated with some of the most common diagnoses like the QBP’s for hip fractures changes that culture. So, I think there’s a little bit of a dichotomy right now happening within the one system as to... what’s the priority and how to stratify them if you will, because there’s multiple priorities here
Workflow	Lack of bed availability, community resources and funding	19	66	Environmental context	Administrators, physicians, nurses and other health professionals	Waiting for a bed. I’m definitely in for that because then it just means that, unfortunately it means that we can do more but... which means more people are falling, which is, again, a little scary
Workflow	Need for extended hours and increased staff (including on weekends)	16	55	Environmental context	Administrators, physicians, nurses and other health professionals	Probably the biggest area is we’re not doing seven-day-a-week admissions here in Geriatric Rehab. And so, you know, I just talked about if we have empty beds. So if we go in on a Friday afternoon, it’s four o’clock, we have two empty Geriatric Rehab beds, they’re not going to be filled ‘til Monday. And so, you know, if you had late day referrals from [the hospital] or... there’s nobody at [the hospital] to refer on the weekend, so it’s the same issue at [the hospital]... is a lot of the work stops on the weekend. So both organizations operate very much kind of five days a week, and so that’s... to me, if we were really going to try to get a target of moving people within seven days, both of our respective organizations would have to more seven-day-a-week... I know the orthopaedic surgeons will say they operate seven days a week. They do. But the system doesn’t operate seven days a week
Admission criteria for geriatric rehabilitation	Need clarity about the geri-rehab program	6	21	Knowledge	Administrators and physicians	I think there’s a bit lack of understanding from all the acute care facilities about what [geriatric rehabilitation] can and can’t do because we’re a subacute hospital
Workflow	Making decisions about the referral processes takes a lot of time	6	21	Memory and decision-making	Administrators, nurses and other health professionals	Other times it’s more complicated. They have a lot of comorbidities, there’s a lot of family issues, there could be caregiver burnout, there could be... they might not be able to go home, they need to look into retirement homes. So it depends. It’s very situational specific
Admission criteria for geriatric rehabilitation	Lack of control of the referral process to geriatric rehabilitation	5	17	Professional roles	Administrators, nurses and other health professionals	I don’t have any control on when they’re referred. Right? I have zero control on if they’re referred day three post-hip fracture, or day five. The only control I have is when I get them
Standardized pathway	Inappropriately transferring complex patients to geri rehab impedes patient progress and causes delays in the system	4	14	Environmental context	Administrators and other health professionals	...maybe we’d be willing to send patients a bit too early. Maybe they’re not just ready yet, or not medically stable. You know, if you send them out early and then, you know, they were just starting to develop a pneumonia, well, you know, you’re impeding the rehab and then you’re sending them to a facility which may not have the resources to deal adequately with that pneumonia
Admission criteria for geriatric rehabilitation	Lack of trust between teams	4	14	Social influence	Administrators, physicians and other health professionals	There’s a lot of hesitancy I think between hospitals to take another patient if, let’s say, somebody else was saying, well, the patient is ready because they don’t necessarily know if the patient is truly ready according to their standard or if they’re just trying to move the patient out of their room, move them out of the system
Education for patients/families	Emotional factors affect my care delivery	4	14	Emotion	Nurses and other health professionals	But yeah, I do have worries because I do have a life outside, right? Everybody brings, you know, have their own many things to go through. So, it does affect at times, I suppose, like, patients, or I have no patience to... for, you know... I have no patience for physicians or things that are not giving me what I want. I want it now and I want it now. So that is one thing, right? I have more patience with my patients, but I don’t necessarily have much patience with everybody around. But that is something that I bring that is part of my life and part of my, kind of, irritation through the system because I live it at home and I see it here and it’s unfair and... but that’s my, my thing... so, yeah, it comes in
Workflow	Unknown about how referral process would look like in the electronic medical record	4	14	Memory and decision-making	Administrators and physicians	There’s Epic now so we’ll have to put it into that. Like you have to consider that. There may be the outliers that don’t fit into the protocol…
Standardized pathway	Discharge destination is influenced by physio and social worker’s recommendations	3	10	Social influence	Administrators and other health professionals	Social work, they would influence it based on whether or not they... if there was a discharge disposition issue, whether they could resolve that or not. ‘Cause if it can’t be resolved and they have no discharge disposition, then that’s going to completely stop the referral altogether
Standardized pathway	Lack of awareness exists about the referral process for staff on other units	2	7	Skills and behavioural regulation	Administrators and physicians	Like I mentioned, on other units where they’re not used to caring for hip fracture patients, it’s definitely more challenging from... even from speaking to the physios, the managers on the units, and the nurses, to set the right expectations
Admission criteria for geriatric rehabilitation	Bed availability in geri rehab is an issue	2	7	Belief about consequences	Administrators and physicians	It’s not predictable what the outcome will be because it’s not just dependent on strict clinical criteria. It depends on what their bed status is
Admission criteria for geriatric rehabilitation	Challenges with patients with cognitive decline	2	7	Belief about consequences	Administrators and physiotherapist	So I think the guidelines are hard to follow, depending on their cognitive level
Admission criteria for geriatric rehabilitation	Subjective selection of patients for geri rehab	2	7	Environmental context, memory and decision-making	Physicians	I think in the past... and maybe it’s gotten better over time, but in the past, we felt that they – some rehab services would come along and take the easy patient who probably would be able to get home in a day or two, and they would take their to short term rehab, for example, and keep them there for 10 or 12 days rather than taking the challenging, more complex patient, that probably needs it more to get out of the building. That may not be as specific to geriatric rehab, but that’s what we see sometimes
Provider culture and influence	Lack of knowledge about roles and responsibilities for the referral process	2	7	Professional roles and knowledge	Administrators and other health professionals	And for the other professions, I’m not sure what the requirements are
Workflow	Re-educating patients on process in geriatric rehab is challenging	2	7	Environmental context	Administrators and other health professionals	So, you know, whether it’s people getting used to doing something a different way, uh, and having to be reminded several times until it becomes a standard practice, and there will be people who may feel like it’s better…
Workflow	Feeling overwhelmed	2	7	Emotion	Administrators and nurses	We’re constantly hearing about patients being sent home who are over the age of 65 after a hip fracture because they could get them home. And then they end up back in acute care several months later, perhaps with another fracture and you really do wonder, had they come for rehab after their first hip fracture
Workflow	Delays in surgery affects patient’s post-surgery	2	7	Environmental context	Nurses and other health professionals	I think the difficulty is sometimes when the patients are waiting a while for their surgery and then when finally get to see them, they are, you know, more sore because they’ve been in bed for so long or, you know, weaker because they’ve been in bed for five days and they lost a percentage of their muscle mass

^a^Frequency within theme (N).

**Table 5.** Enablers to the Implementation of Best Practices for the Management of Hip Fracture Patients Transitioning from Acute Care to Subacute Care (n = 17).

Category	Overarching enablers across domains	N^a^	%	Domain	Who said it?	Example quote
Education for patients/families	Clear communication with patients and families	27	93	Professional roles, skills and social influence	Administrators, physicians, nurses and other health professionals	So I think, again, if we could, if we can all say the same thing, if we could... well, say the same thing... not create expectations that are... can’t be met. Trying... again, just trying to say the same message from the beginning through to the surgery, through to the... on the unit, through to rehab, look at the person as a whole
Standardized pathway	Benefits of geriatric rehabilitation for patients	24	83	Reinforcement and optimism	Administrators, physicians, nurses and other health professionals	I think it’s really important because it’s addressing not only minimizing acute care, length of stay and getting the person to the right place at the right time, but it’s also looking to try and approach it from not just siloed health care, but what does the whole person need to get them to the best place so that they are benefitting the most as an entire person, so that they’re going back out and they have that cognitive as well as physical, support network, essentially, established, and, and support is as best possible
Standardized pathway	Need for education and resources in order to properly use the pathway	15	52	Behavioural regulation and beliefs about consequences	Administrators, physicians, nurses and other health professionals	System-wide approaches often involve a lot of players, so there needs to be a lot of education and a lot of support around sustainability of any new initiatives that come into place to ensure that everyone is doing the same thing, everyone’s on the same page, and to ensure that it can be continued into the future
Education for patients/Families	Providing patients and families with resources and setting clear and consistent expectations	14	48	Behavioural regulation and professional roles	Administrators, physicians, nurses and other health professionals	I mean certainly information, education for the patients before they arrive, and then on admission, we have to do the same thing when they get here, standardize their expectations. Of course, that falls on to us, yeah, I think that would help
Admission criteria for geriatric rehabilitation	Focus on factors to diminish delirium	13	45	Belief about consequences	Administrators, physicians, nurses and other health professionals	But I think from a medical point of view, if they were able to identify or prevent delirium from happening post-op in this population, I think we’d be ahead of making sure that these people would transition through the system a lot faster
Admission criteria for geriatric rehabilitation	Knowing about geriatrics and the geri-rehab program would be useful	12	41	Environmental context, knowledge and belief about consequences	Administrators, physicians, nurses and other health professionals	If they, you know, if people can be, you know, for example, from Acute Care, having their team be at physio, OT, nurses, physicians... come over to see what our program is about. And for us to go over to their side with our care team to be able to see how things work there. I think that helps with communication and building understanding
Workflow	Need for all necessary information about the patient	11	38	Behavioural regulation	Administrators, physicians, nurses and other health professionals	So, just having the proper information would make my life easier, and then trying to get them not all out of it from surgery
Admission criteria for geriatric rehabilitation	Confidence to identifying geri-rehab candidates and potential medical concerns or barriers that might be an issue for geriatric rehabilitation	11	38	Belief about capabilities	Administrators, physicians and other health professionals	I think it’d be pretty easy to determine very early on when someone is going to be appropriate and that would decrease the wait time
Standardized pathway	Data would be helpful to encourage use of pathway	10	35	Reinforcement, behavioural regulation and skills	Administrators, physicians and other health professionals	...if you were to ever look down the road and really want to maybe guarantee that an orthopaedic patient gets in in so many days, then you would have to absolutely know, every day, I’m going to be counting on three hip fracture patients. And then you might consider streaming them to certain beds, you know, maybe having a, like a certain triage line. But you can’t do that when you’re not, when you don’t have that steady state
Workflow	Resources are adequate	9	31	Environmental context	Administrators, physicians and other health professionals	I don’t know that we need more resources. I think we have a fairly good setup right now
Workflow	Patients and families influence the care I provide	8	28	Social influence, memory and decision-making	Administrators, physicians, nurses and other health professionals	We need to ensure that when, you know, we’re making a decision about rehab that obviously the patient’s included in that and that communication happens first from the acute care team to discuss rehab and some of the various options that exist for rehab
Standardized pathway	Need everyone to buy in	6	21	Behavioural regulation	Administrators, nurses and other health professionals	The pathways only good in that the whole circle, the whole circle of care, both at discharge, community-based, and pre-, you know pre-the acute level, that we all work together. If one piece is going to be able to opt out, that wouldn’t make sense and would not make the pathway very efficient
Admission criteria for geriatric rehabilitation	Helpful to identify candidates early for geri rehab	6	21	Behavioural regulation	Administrators, physicians and nurses	I think we could probably identify most of these, you know, within five minutes of them arriving in the emergency department. So, a patient that comes in, do it objectively through frailty score, through just, you know, review of their medical comorbidities
Workflow	Need to be made aware of specific restrictions	5	17	Behavioural regulation	Administrators, physicians and other health professionals	the total hip replacement surgery, depending on the location or the, you know, how, if they do an anterior approach, posterior approach, there are restrictions that come automatically with that patient. So I’m thinking the referral initial package should include that, you know, that information. Often it says that if their weight-bearing is tolerated, but they don’t mention anterior precautions or posterior precautions and the real crucial piece is, how long do those precautions have to be maintained. That is often missing from the package and we won’t get that info until they go back to visit Ortho, and if they don’t visit Ortho, then we don’t know
Admission criteria for geriatric rehabilitation	Flexibility in the eligibility criteria	4	14	Behavioural regulation	Administrators and physicians	I think there needs to be the, you know, I think, like, they call it the “80/20 rule”, right? Where, this is the framework, and these are the guidelines. But if you have somebody who really, for whatever reason, whether it be medical or they have a post-op, whatever, you’re not going to go see your post-op hip fracture day three who’s still in the ICU, right? I think there needs to be some room for flexibility if someone really is not appropriate to be referred that quickly
Standardized pathway	Reassessment of declined patients is needed	2	7	Behavioural regulation and belief about consequences		With the surge funding, what they’ve done is increased a position where there’s somebody that’s actually following cancelled referrals. So when a referral comes in but the patient is not medically stable enough to be considered for rehab, or declined from rehab due to outstanding issues, typically what happens is the referral’s cancelled and we stop following. But with the surge money, what they’re doing is having somebody follow up. And so if there’s a couple of outstanding tests that need to be done, and so they’re accepted but not ready, the goal is to have that person follow up to ensure that those tests are done in a timely manner, and then that message is communicated back to rehab to get them from “Accepted... not ready” to “Accepted... ready” and over and out of the hospital and into rehab where they need to be. As well as any referrals that get cancelled due to medical stability, following up so that we are aware as soon as they become medically stable, to reinitiate that referral and get the process started again rather than have it kind of fall through the cracks or not be noticed for a couple of days before the referral’s initiated
Admission criteria for geriatric rehabilitation	Helpful to identify candidates who deviate or deteriorate in order to revoke the referral to geri rehab	2	7	Behavioural regulation and, belief about consequences	Administrators and physicians	But I think we could probably identify those fairly early on, during admission and sort of early post-operatively if we think that they’re going to be longer. I think they’d be... usually it’s fairly easy to identify, but sometimes, you know, unpredictable things happen

^a^Frequency within theme (N).

**Table 6.** Conflicting Themes to the Implementation of Best Practices for the Management of Hip Fracture Patients Transitioning from Acute Care to Subacute Care (n = 4).

Category	Conflicting themes (barrier or enabler)	N+	%	Domain	Who said it?	Example quote
Standardized pathway	Concerns with a standardized approach (barrier)	25	86	Belief about consequences and optimism	Administrators, physicians, nurses and other health professionals	I think the negative aspect would be, you know, every individual is different. You know, every person is different and, you know, I don’t see there would be a huge... but some person may not like that and they say, “Why are we all... it’s not like a cookie-cutter recipe where everybody has to follow the same protocol”, you know? There are always exceptions. There could be comorbidities that could impact...
Standardized pathway	A standardized pathway would be beneficial (enabler)	28	97	Behavioural regulation, beliefs about consequences and intention	Administrators, physicians, nurses and other health professionals	Yeah, so exactly what I said, and you won’t miss the average individual and they’re all going to get the same level of care. It’s along the same line as a clinical pathway where it’s based on evidence-based practice and you, everybody... that standard of care hits 80 percent of the population and its good care
Workflow	Poor communication and collaboration between acute care and geriatric rehabilitation (barrier)	9	31	Social influence	Administrators, physicians, nurses and other health professionals	I think, especially because different care providers are from different hospitals, they may not understand how each other-- team functions and I think that would be a challenge but not an impossible barrier to overcome either
Workflow	Communication and collaboration between acute care and geri rehab is well established (enabler)	29	100	Social influence, skills and knowledge	Administrators, physicians, nurses and other health professionals	So I think if we were to ask what would be good in our system, it would be having that really close collaboration between Geriatrics and our team, which, luckily, we have, but still, I think it’s important to mention that that’s an ideal place to be. We really connect very closely
Workflow	Referring patients to geriatric rehabilitation is not my top priority (barrier)	7	24	Goals	Administrators, physicians and other health professionals	Keep in mind that sometimes I have to de-prioritize assessing the fitness of somebody for rehab today if I am worried about another patient on the unit having, like, an acute decompensation, low pressure code needs to be called. So, those are things that factor in in a little bit
Workflow	Referring patients to geriatric rehabilitation is my top priority (enabler)	21	72	Goals	Administrators, physicians, nurses and other health professionals	It’s very important because we need to keep our flow, because if we don’t have flow then we don’t have beds for the next surgery. So, it’s very important to get them moving. It’s important for the patient to get moving to the right location so they can work on strengthening and returning home
Admission criteria for geriatric rehabilitation	Not aware of the evidence that supports the guidelines (barrier)	15	52	Knowledge	Administrators, physicians and other health professionals	I haven’t read the actual evidence, like specific publications or any scholarly articles on it or anything. I just know from hearsay, so word of mouth
Admission criteria for geriatric rehabilitation	Awareness of exiting hip fracture guidelines (enabler)	29	100	Knowledge	Administrators, physicians, nurses and other health professionals	We follow a variety of guidelines as to whether patients are able to participate in rehab. We take our recommendations through our physiotherapists. Once they have seen them and assessed the patient, the patient has to be able to participate in a rehab program. They have to be over the age of 65, they have to be medically stable

#### Barriers

Five categories of barriers were identified: (A) standardized pathway, (B) workflow, (C) admission criteria for geriatric rehabilitation, (D) education for patients/families and (E) provider culture and influence. The top 3 barriers were (1) competing demands (n = 24); (2) a lack of bed availability, community resources and funding (n = 19); and (3) the need for extended hours and increased staff (including on weekends) (n = 16).

#### Enablers

Four categories of enablers were identified: (A) workflow, (B) admission criteria for geriatric rehabilitation, (C) standardized pathway and (D) education for patients/families. The top 3 enablers were (1) clear communication with patients and families (n = 27), (2) awareness of the benefits of geriatric rehabilitation for patients (n = 24) and (3) the need for education and resources in order to properly use the pathway (n = 15).

#### Conflicting themes

Some themes (n = 4) were seen as both a barrier or an enabler depending on the interview. Some key informants had concerns with a standardized approach (barrier) (n = 25) and some thought a standardized approach would be beneficial (enabler) (n = 28). Some key informants felt it could be both a barrier and an enabler depending on the situation. All key informants felt that communication and collaboration between acute care and geriatric rehabilitation was well established (enabler) (n = 29), but some felt that poor communication and collaboration between acute care and geriatric rehabilitation was a concern in certain situations (barrier) (n = 9). There were conflicting views with respect to referring patients to geriatric rehabilitation as their top priority (enabler) (n = 21) or not referring patients to geriatric rehabilitation as their top priority (barrier) (n = 7). All key informants were aware of existing hip fracture guidelines (enabler) (n = 29); however, some key informants were not aware of the evidence that supported the guidelines (barrier) (n = 15).

### Patients’ and Informal Caregivers’ Interviews (n = 9)

#### Barriers

Based on the perspective from patients’ and informal caregivers’ barriers included: (1) a lack of care coordination between the orthopaedics units and the geriatric rehabilitation service, (2) overcoming some of their own specific challenges during their transition, (3) gaps in the information they received prior to discharge, (4) not knowing what questions to ask because of the lack of information provided and (5) a lack of resources (Table 7).

**Table 7.** Barriers Identified by the Patients and Informal Caregivers (n = 5).

Barrier	Description	Example quotes
A lack of care coordination between the orthopaedics units and the geriatric rehabilitation service	lack of staff accountability/no clear ownership of the referral process not being involved in the decision-making process feelings of overwhelmingness with the referral process	‘And so that the hospital didn’t really have any authority in terms of when that process... when that assessment would take place, when the transfer would take place, when the admission would take place. And by that I mean several times I was asked to be there at early in the morning, to be there to provide the information to the person assessing from [geriatric rehabilitation], and was stood up. ...and I’m thinking, is that too much to ask, to have somebody own this process? Like, yeah, no one’s just... there’s just... no one’s taking responsibility’. Caregiver #35 ‘It was definitely their recommendation, based on their assessment. And we have not been explained what either program is like, or given the information in order to make any kind of informed choice’. Caregiver/Patient #33 ‘Like, just the whole admission process there. Like, the application process and everything. So that was a bit frightening’. Caregiver #35
Overcoming some of their own specific challenges during their transition	medical complications challenges with medication management delays due to the wait for test results or delirium	‘Quite frankly, I was such a basket case. I was basically psychotic because of the problem with the pain medication and the sleep deprivation that I was not really operating as a normal person’. Patient #19 ‘So that, that transition was not well-handled in terms of the medication piece, which I found quite shocking, frankly’. Caregiver #35 ‘...I wasted a couple of days being in limbo there. Because can you imagine, you get a DNA test today, you can know... who your father is tomorrow... but me? It was a bowel movement test, they couldn’t get it. After two, after a week, and a half. I can’t get over it’. Patient #28 ‘At the time that the application was done. I think she’s probably a little bit behind because of the confusion. So it’s possible that now she might qualify for the [geriatric rehabilitation] program if they reassessed her? But at the time that they did the assessment…’ Caregiver/Patient #33
Gaps in the information they received prior to discharge	Lack of information about the Plan of care/length of stay Geri-rehab program from ortho staff inconsistent information from staff	‘I had no idea what would happen. Absolutely no idea. No idea’. Patient #21 ‘And I found that the General told me everything that was going to happen there, at their place, but they could not provide information about this centre here, the rehab centre. They could not, because they didn’t know what was going to happen to me...they couldn’t answer my questions, so I had to wait until I was here’. Patient #28 ‘But the surgeons... we spoke with the surgeons before the operation and everything was outlined there. But there was never any idea that was given in terms of...this is approximately how long she’s going to be in the hospital’. Caregiver/Patient #33
Not knowing what questions to ask because of the lack of information provided	Not knowing what to ask Simply accepting the advice given Being uncomfortable asking questions	‘...there was, I think, definitely for both of us, there were some question marks, but we didn’t know what question to ask. So when you’re told something, you just say, Okay. I’m good to go with it....at what level you get... and who you should pay attention to the advice you get. Just that sometimes you have questions but you don’t want to ask them, so it is a matter that somebody should be giving you more information? Or is a matter that, well, you just throw things out there and see what happens?’ Patient #38 ‘ Again, because it’s new, I really... you don’t know what you don’t know. So I didn’t know what questions to ask’. Caregiver/Patient #33 ‘But maybe I just haven’t asked the questions that need to be asked’. Patient #34
A lack of resources	Lack of equipment and geri-chairs Lack of staff availability	‘But our, yeah, so our hope was that we’d be, you know, getting a bit, getting more physiotherapy, certainly’. Caregiver #35 ‘That’s... and then the lack of equipment. They didn’t even have a Geri chair for her to sit in. And we’re told at the Orthopedic Department, she needs to sit down, she can’t just be lying in her bed’. Caregiver #35 ‘I’m not sure if the staff were happy where I was just because of overload. That’s about all I can say that way’. Patient #19

#### Enablers

Despite these barriers, patients were generally pleased with the transition from orthopaedics to geriatric rehabilitation. A participant said:

“One day I woke up, they said that before 11 o’clock somebody’s going to pick you up. So I sat in a chair, somebody fed me, medicated me, dressed me. I had only to wait... they even packed my things. When the stretcher came on, they just put me on the stretcher and my things underneath or some... on top of me... they drove me here. That was perfect.” (Patient #28).

Overall, the participants described the transition as being good and being pleased with the care received.

## Discussion

### Summary of the Findings

In this study, we used a behavioural theory approach to identify the barriers and enablers likely to influence the implementation of best practices for early identification, referral and access to geriatric rehabilitation post-hip fracture. This comprehensive and systematic approach identified barriers and enablers in the following categories: standardized pathway, workflow, admission criteria for geriatric rehabilitation, education for patients/families and provider culture/influence, all of which that have the potential to inform a future behaviour change intervention.

Barriers identified by clinicians and administrators were related to organizational and system factors such as competing demands for their time to perform other tasks or activities, a lack of bed availability, a lack of community resources, a lack of funding and the need for extended hours and increased staff including on weekends. Enablers were primarily related to patients’ and informal caregivers’ needs, and included items such as clear communication with patients and families, awareness of the benefits of geriatric rehabilitation and the need to receive adequate education and resources to properly use the pathway. Although, patients and informal caregivers were generally pleased with the transition from orthopaedics to geriatric rehabilitation, they too described some organizational level barriers such as a lack of care coordination between the orthopaedics units and the geriatric rehabilitation service, gaps in the information they received prior to discharge and a lack of equipment and staff resources. They also provided their personal experiences with their care transition with common themes including not knowing what questions to ask because of the lack of information provided and having to overcome some medical-related complications and delays during their transition.

Understanding these barriers and enablers will strongly support the future development of an evidence-based intervention to improve early identification, referral and access to geriatric rehabilitation post-hip fracture. The transition from acute to subacute care requires input from multiple team members, and consent from patients and/or families. The variety of responses elicited demonstrated the need for careful planning of any intervention in order to engage all stakeholders and to effect long-lasting change.

### Comparison to Previous Research

Despite the ample evidence attesting to the benefits of geriatric rehabilitation programmes for post-hip fracture patients, our study reinforced the fact that a gap in knowledge exists regarding the barriers and enablers to better care transitions between acute and subacute care for geriatric hip fracture patients. This study also highlights the variation in practice for this population. The variation in practice impacts patients’ outcomes as well as overall health care delivery and costs. One systematic review demonstrated that inpatient rehabilitation specifically targeted at geriatric patients improved outcomes related to functional status and decreased mortality rates.^19^ Other studies found that geriatric hip fracture programmes were associated with health and social service savings and were more effective than usual care in reducing length of stay,^6,20,26^ improving function^20^ and increasing the rate of return to home after discharge.^20,27^

In a recent study,^28^ the researchers reported that the main barrier to expedite patient discharge was arranging the appropriate placement for patients requiring long-term advanced care with home health nursing or inpatient nursing facilities. In another study, researchers examined the impact of an earlier transfer of hip fracture patients to rehabilitation for ten partnerships between acute care and rehabilitation.^29^ Their results showed that only 2 of the partnerships were able to achieve the target reduction in length of stay, indicating that these care transitions are complex events.^29^ The barriers to earlier transfer to geriatric rehabilitation are similar to the ones in our study. They include contradictory opinions regarding patients’ eligibility for rehabilitation, inefficient hospital system processes and hospital pressures (i.e. occupancy). The study also supports the need for a high degree of collaboration between acute and subacute care to realize change.^29^

Based on the results of our study, we have designed an evidence-based intervention for early identification, referral and access to geriatric rehabilitation post-hip fracture. The pathway will enable all patients if they meet the defined eligibility criteria for geriatric rehabilitation to be accepted and transferred from acute care to subacute care no later than post-op day 6.^1^ The implementation of this pathway is guided by our barriers and enablers analysis. Specifically, we selected the evidence-based behaviour change techniques (BCTs)^23^ that address these barriers and enablers. These selected BCTs include the development of key flags in the clinical pathway, standardized transfer of information between acute and subacute care, high-risk delirium screening and dashboards to provide immediate feedback to clinicians, patient information materials, multidisciplinary workshops, reminders and sustained engagement. We have combined these BCTs into a deliverable intervention that will be evaluated in a future trial for feasibility and acceptability.

### Strengths and Limitations

There are strengths and limitations to our study. We used a theoretical framework to guide our data collection and analysis. This study also included a large variety of clinicians and administrators to get a better insight on the barriers and enablers to the referral to geriatric rehabilitation post-hip fracture. Although we interviewed a total of 38 participants consisting of clinicians, administrators, patients and informal caregivers, it is possible that the participants’ views differ from those who did not participate. However, we did obtain some very important information on what might be the barriers and the enablers to the development and the implementation of the intervention.

## Conclusion

This study identified key barriers and enablers to early identification, referral and access to geriatric rehabilitation post-hip fracture. A better understanding of these barriers and enablers can inform and optimize future implementation strategies so that they are better tailored to the local context and can also inform wider implementation. Overall, addressing these barriers pre-implementation may improve the integration of the standardized pathway into practice.
